# Antimicrobial Stewardship Interventions in Pediatric Oncology: A Systematic Review

**DOI:** 10.3390/jcm11154545

**Published:** 2022-08-04

**Authors:** Edoardo Muratore, Francesco Baccelli, Davide Leardini, Caterina Campoli, Tamara Belotti, Pierluigi Viale, Arcangelo Prete, Andrea Pession, Riccardo Masetti, Daniele Zama

**Affiliations:** 1 Pediatric Oncology and Hematology “Lalla Seràgnoli”, IRCCS Azienda Ospedaliero-Universitaria di Bologna, 40138 Bologna, Italy; 2 Division of Infectious Diseases, IRCCS Azienda Ospedaliero-Universitaria di Bologna, 40138 Bologna, Italy; 3 Pediatric Unit, IRCCS Azienda Ospedaliero-Universitaria di Bologna, 40138 Bologna, Italy; 4 Pediatric Emergency Unit, IRCCS Azienda Ospedaliero-Universitaria di Bologna, 40138 Bologna, Italy

**Keywords:** antibiotic stewardship programs, antifungal stewardship, antibacterial stewardship, antibiotic resistance, antibiotics, pediatric hematology, pediatric oncology

## Abstract

Antimicrobial stewardship programs represent efficacious measures for reducing antibiotic overuse and improving outcomes in different settings. Specific data on pediatric oncology are lacking. We conducted a systematic review on the PubMed and Trip databases according to the PRISMA guidelines, searching for reports regarding antimicrobial stewardship in pediatric oncology and hematology patients. The aim of the study was to summarize the present literature regarding the implementation of antimicrobial stewardship programs or initiatives in this particular population, and provide insights for future investigations. Nine papers were included in the qualitative analysis: three regarding antifungal interventions, five regarding antibacterial interventions, and one regarding both antifungal and antibacterial stewardship interventions. Variable strategies were reported among the included studies. Different parameters were used to evaluate the impact of these interventions, including days of therapy per 1000-patient-days, infections with resistant strains, safety analysis, and costs. We generally observed a reduction in the prescription of broad-spectrum antibiotics and an improved appropriateness, with reduced antibiotic-related side effects and no difference in infection-related mortality. Antibiotic stewardship programs or interventions are effective in reducing antibiotic consumption and improving outcomes in pediatric oncology hematology settings, although stewardship strategies differ substantially in different institutions. A standardized approach needs to be implemented in future studies in order to better elucidate the impact of stewardship programs in this category of patients.

## 1. Introduction

Infections represent a major cause of morbidity and mortality in pediatric patients with cancer [1]. Several aspects differ in terms of epidemiology and management compared to adults. The differences in cancer types and subsequent treatment intensity, co-morbidities, and environmental factors, along with a developing immune system, result in a specific epidemiology of infections in children [2]. Therapeutic protocols often have higher dose intensity compared to those for adults, and this may result in a high incidence and severity of treatment-related complications, making the management of infections a key issue in pediatric oncology [2]. The choice and use of antimicrobial agents present peculiar considerations that need to be considered in this subgroup of patients, including pharmacokinetic and safety profiles, and specific data are not always available [3,4]. Age-related challenges in drug formulation should also be considered, as well as regulatory issues, with many pharmaceutical agents being used in off-label or unlicensed settings [2,5].

The use of broad-spectrum antimicrobial agents and the prompt initiation of therapy are critical in vulnerable immunocompromised children when the suspicion of infection is high [6]. However, the adverse effects of larger-spectrum drugs should be considered, including *Clostridioides difficile* infection, gut microbiota dysbiosis, and the emergence of antimicrobial resistance [7,8,9,10]. In particular, the rise in resistance of bacterial pathogens is a major concern, and new concepts for antibiotic treatment are urgently needed in order to minimize the unnecessary administrations and optimize antibiotics use [1]. More recently, the optimization of antifungal therapy has been recognized as a critical issue in the management of pediatric oncological patients. A more refined antifungal selection could improve patient outcomes, reducing toxicities and drug interactions [11]. Moreover, limiting the inappropriate antifungal use could reduce the recent rise in multidrug-resistant microorganisms [12].

Antimicrobial stewardship programs (ASPs) are defined as “coordinated interventions designed to improve and measure the appropriate use of antimicrobial agents by promoting the selection of the optimal drug regimen including dosing, duration of therapy and route of administration” [13]. A growing body of evidence demonstrates that ASPs reduce antibiotic overuse while improving patient outcomes in different pediatric settings [14]. Despite the increasing implementation of pediatric ASPs, data on the impact of ASPs in the oncology wards are limited [15]. Physicians’ concerns regarding narrowing or discontinuing antimicrobial therapy in high-risk pediatric oncological patients can limit the implementation of ASPs. Demonstrating the impact of ASPs in these patients is therefore a critical issue. In adults, antibiotic stewardship interventions for the management of febrile neutropenia (FN) in hematological malignancy units effectively reduced carbapenem use, decreased vancomycin-resistant *Enterococcus faecium* colonization and infection, and reduced daptomycin prescription with no change in mortality [16].

In children, the evaluation of clinical outcomes associated with ASP interventions in high-risk pediatric oncology patients is lacking. In this study, we aim to provide an up-to-date systematic review regarding the implementation of antimicrobial stewardship interventions in pediatric oncology, summarizing the present literature and providing insights for future investigations.

## 2. Materials and Methods

The present systematic review was conducted according to the Preferred Reporting Items for Systematic Reviews and Meta-Analyses (PRISMA) guidelines [17]. Electronic databases—namely, PubMed (https://pubmed.ncbi.nlm.nih.gov accessed on 17 January 2022) and Trip (https://www.tripdatabase.com accessed on 17 January 2022)—were searched on 17 January 2022 to identify relevant studies. The following string was used to perform the literature search: (antimicrobial stewardship OR antibacterial stewardship OR antibiotic stewardship OR antifungal stewardship OR antimicotic stewardship OR antiviral stewardship) AND (oncology OR hematology OR onco-hematology OR haematology OR onco-haematology OR cancer OR bone marrow transplant* OR BMT OR stem cell transplant* OR SCT OR hematopoietic transplant* OR haematopoietic transplant* OR hematopoietic stem cell transplant* OR haematopoietic stem cell transplant* OR HSCT) AND (children OR childhood OR pediatric). The search was restricted to English-language studies involving human subjects assessing the implementation of antimicrobial stewardship programs or initiatives, regardless of type, in pediatric patients with hematological/oncological disease and/or receiving hematopoietic stem cell transplantation. The types of studies considered eligible for this systematic review were observational studies—both retrospective and prospective—and randomized clinical trials. Case reports and other systematic reviews or meta-analyses were excluded. Clinical, pharmacological, and microbiological outcomes of stewardship initiatives were assessed, and are reported in this systematic review. Studies that performed only antibiotic surveillance or surveys on antibiotic use, as well as studies that measured the clinical effects of antimicrobial agents, were excluded. We included studies that reported data regarding only pediatric oncology patients, or that separately described the information regarding this subgroup of patients.

Two authors (E.M. and F.B.) screened the results of the literature search and independently identified potentially eligible studies by assessing the full titles and abstracts. The same reviewers evaluated the full texts of potentially relevant studies for inclusion, and consulted the reference lists of previously published primary and secondary papers to manually search for additional relevant studies. Any disagreement regarding eligibility and inclusion in the systematic review was resolved through discussion and consensus between the two authors. If consensus was not reached, the opinion of a third reviewer (D.L.), who acted as a “blind” final arbiter, was requested. Corresponding authors or investigators were contacted for studies with incomplete data in order to obtain additional information if needed.

We performed the quality assessment of included studies using the Strengthening the Reporting of Observational Studies in Epidemiology (STROBE) statement [18]. The STROBE statement is a 22-item tool designed to evaluate the quality of observational studies. Items are associated with different sections of an article, such as title and abstract (item 1), introduction (items 2 and 3), methods (items 4–12), results (items 13–17), discussion (items 18–21), and other information (item 22 for funding). Eighteen items are identical for the three different study designs, whereas four items (items 6, 12, 14, and 15) are differentially intended for specific study types. The STROBE statement does not provide scoring stratification itself, but it implies that the higher the score, the higher the quality of the study is considered. As previously described [19], we propose 3 score thresholds, corresponding to 3 levels of quality assessed: 0 to 14 is considered poor quality, 15 to 25 is intermediate quality, and 26 to 33 is good quality. Two authors (F.B. and D.L.) performed the quality assessment considering all of the items of the STROBE statement. If consensus was not reached, the opinion of a third reviewer (E.M.), who acted as a “blind” final arbiter, was requested.

## 3. Results

### 3.1. Literature Search

**Figure 1:**
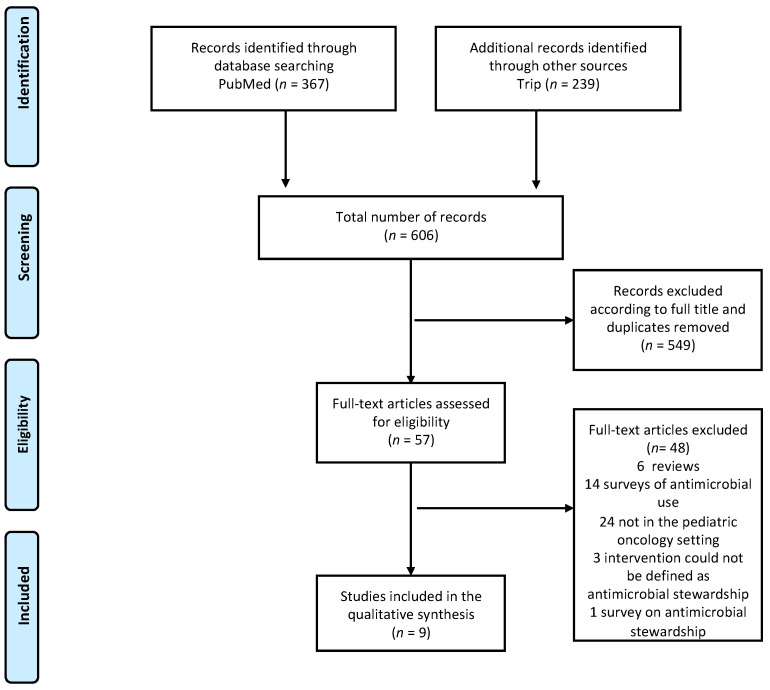
PRISMA flow diagram of the study.

The literature search strategy identified 606 records (367 in PubMed and 239 in Trip). The number of potentially relevant references identified by full titles was 57 (Figure 1). Among the papers assessed for eligibility, 6 were excluded because they were reviews, 14 because they reported surveys on antimicrobial use, and 24 because the data in the studies did not include pediatric oncology patients or did not separately report the information regarding this subgroup of patients. Three studies among the fifty-seven assessed were excluded from the systematic review because the intervention described could not be defined as antimicrobial stewardship. One more study was excluded because it did not describe analysis of antimicrobial stewardship implementation or interventions, but was a cross-sectional survey analyzing different aspects of ASPs [20]. No randomized studies were found in the literature. Of the nine observational studies included in the qualitative synthesis, three addressed interventions regarding antifungal stewardship, while five analyzed programs or initiatives of antibiotic stewardship. One paper included both antibacterial and antifungal stewardship interventions. In the following sections, we present evidence regarding the implementation of ASPs regarding both antibacterial and antifungal stewardship initiatives.

### 3.2. Antibacterial Stewardship

Six studies investigated antimicrobial stewardship interventions specifically regarding antibacterial therapies in pediatric oncology and hematology patients. One paper studied the impact of an ASP without a comparison with the pre-intervention phase [21]. Five works analyzed the efficacy of different ASPs, investigating the differences between two phases—before and after the introduction of specific interventions (pre-intervention and post-intervention phases). These analyses were performed by the comparison of different parameters regarding antibiotic use and appropriateness (Table 1). Four of them also included an analysis of safety with comparison of clinical outcomes before and after the intervention. Quality assessment of the included studies is reported in Table 1, and extensively in the Supplementary Materials.

Dhanya et al. studied the impact of an ASP without a comparison with the pre-intervention phase. This multicenter study evaluated the introduction of weekly rectal swab surveillance cultures as a resource to identify gut colonization with extended-spectrum beta-lactamase (ESBL)-producing *Escherichia coli* or Klebsiella pneumoniae carbapenemase (KPC)-producing organisms, and to guide empirical antibiotic therapy in allo-HSCT performed mainly for nonmalignant disorders. The authors observed that choosing empirical antibiotic therapy based on rectal swab cultures is not justified, with the clinical response not being correlated with the antimicrobial susceptibility tests. Furthermore, colonization status showed no correlation with clinical outcomes [21].

Wattier et al. reported the impact of a multistep intervention including updating of institutional guidelines on FN, and the introduction of an ASP. The authors showed a significant reduction in second-line antibiotic use (tobramycin and ciprofloxacin), measured as days of therapy (DOT)/1000 patient-days after both interventions. A more sustained long-term effect was achieved with the introduction of stewardship compared to updating of guidelines, demonstrating major benefits of active and ongoing interventions over single-phase passive interventions [22].

Horikoshi et al. similarly demonstrated a reduction in IV antibiotic use in DOT/1000 patient-days after the introduction of an ASP. In particular, they found that the DOT of cefepime, piperacillin/tazobactam, meropenem, vancomycin, liposomal amphotericin B, and fluconazole decreased by 20%, 45%, 57%, 38%, 85%, and 44%, respectively (*p* < 0.05). A further analysis of this study included the appropriateness of empirical therapy (% of microorganisms susceptible to the empirical agent isolated in blood culture with), which did not show any difference after the intervention. Furthermore, the authors observed a reduction in costs, with savings of USD 59,905 annually [23].

Hennig et al. focused on the use of gentamicin pre/post-guideline and ASP introduction. The authors found a significant decrease in gentamicin use. Furthermore, the analysis of gentamycin’s appropriateness demonstrated that the percentage of Gram-negative infections appropriately treated with gentamicin increased concurrently with decreased gentamicin use in infections without a positive blood culture. The authors also described an increase in the use of therapeutic drug monitoring of gentamicin, as well as a decrease in inappropriately prolonged use of the antibiotic. It should be noted that no safety analysis was available for this study [24].

Karadikar et al. analyzed the impact of changing guidelines regarding vancomycin use on antibiotic administration. New febrile neutropenia guidelines recommended intravenous vancomycin in all high-risk patients for 48 h at the beginning of the episode, with rapid discontinuation in the absence of positive blood culture or compatible clinical symptoms. Updated antibiotic discontinuation criteria included appearing well, being afebrile for more than 24 h, and absolute neutrophil count > 200/mmc. After the update, a significant decrease in overall vancomycin use was observed (DOT/1000 FN days from 2297 to 1758; median empiric intravenous vancomycin days within an episode from 164 to 115; *p* < 0.01). A reduction in the incidence of vancomycin-resistant Enterococcus infections/1000 patient-days was also demonstrated (from 2.53 to 0.90; *p* = 0.02). An increasing adherence to the protocol throughout the study period was also reported [25].

Olson et al. more specifically investigated home intravenous (IV) antibiotic use for febrile neutropenia after hospital discharge, before and after the implementation of internal guidelines. The goal was to change practice from using IV antibiotics after hospital discharge to the use of step-down oral therapy with levofloxacin for most children with FN until absolute neutrophil count > 500. Discharge criteria were determined by the treating physician, and generally included the following: negative blood culture for 24–48 h, afebrile for at least 24 h, and anticipated neutropenia < 7 days. The objectives of this study were to determine the impact of these guidelines on home IV antibiotic use, and to evaluate the safety of their implementation. A significant reduction was noted in adjusted multivariable regression analysis regarding the percentage of patients discharged with intravenous antibiotics (adjusted risk ratio of 0.07, with a 95% confidence interval of 0.03–0.13), along with increased home oral levofloxacin use (adjusted risk ratio of 13.5, with a 95% confidence interval of 7.8–24.1). The authors also found no evidence that the change in practice led to increased treatment failure, *C. difficile* infections, or mortality rate. In this study, multiple secondary outcomes were analyzed in order to investigate the clinicians’ concern for treatment failure after the intervention, including the percentage of patients who received intravenous antibiotics or were tested with a blood culture within 7 days after the discharge [26].

### 3.3. Antifungal Stewardship

Four studies evaluated antifungal stewardship programs in pediatric cancer patients. While one study was a survey, the other three included single-center studies comparing clinical outcomes before and after the introduction of antifungal stewardship programs (Table 2). The quality assessment of the included studies is reported in Table 2 and extensively in the Supplementary Materials.

Mendoza-Palomar et al. reported the results of the PROAFUNGI study—an observational, prospective, single-center survey reviewing the prescriptions of antifungal drugs in a tertiary care pediatric hospital. An antifungal stewardship program was applied for evaluating the appropriateness of the prescription to uncover points of improvement in antifungal use. The majority of the prescriptions were for cancer patients (45.5%)—most of them having undergone HSCT (60%)—and regarded liposomal amphotericin B (46.2%) and posaconazole (17.7%). Interestingly, most of the prescriptions were in a prophylactic setting (63%). The appropriateness analysis revealed that 11% of the prescriptions were considered non-optimal—mostly for the lack of indication (58.3%)—and this was found particularly in cancer and HSCT settings. The authors concluded that the main reason for the inaccuracy of antifungal prescription was the lack of a correct antifungal stewardship program [27].

Horikoshi et al. reported the application of an antibiotic and antifungal stewardship program in a single-center hematology–oncology and HSCT ward. Stewardship interventions consisted of different actions, including prior authorization of drug prescription, prospective audit with feedback by pharmacists and infectious disease specialists, ordering assistance for prescribing physicians with therapeutic drug monitoring by pharmacists, and a weekly clinical luncheon meeting of hematology–oncology and infectious diseases physicians. After the application of the program, the prescription of antifungal drugs such as liposomal amphotericin B and fluconazole decreased significantly, by 75% and 41%, respectively, with the total cost of all antifungal agents decreasing by 20% [23].

Similarly, Santiago-García et al. applied an ASP and assessed its impact on antifungal prescription. An expert team was established including pediatric hematology–oncology and infectious disease specialists. After producing a consensus protocol, a training program was held by the team, and a self-assessment questionnaire was distributed among physicians to assess their knowledge. After the application of the program, the percentage of inadequate prescriptions decreased significantly, by 9.9%. Very interestingly, the self-assessment questionnaire responses revealed a significant increase in knowledge after the course, which remained stable after 12 months [28].

Amanati et al. specifically analyzed the impact of an antifungal stewardship program in a single institution on the local epidemiology of *Candida* species. Similarly to the other reports, the antifungal stewardship program consisted of several actions aiming at guaranteeing appropriate treatment of the suspected IFDs, appropriate antifungal prescription, and a non-medical approach to prevent fungal infections. First, after the application of the program, the authors reported a statistically significant reduction in *C. albicans* strains resistant to azoles—particularly fluconazole and caspofungin. This was also achieved by a reduction in fluconazole use and a consequent reduction in drug expenses [29].

## 4. Discussion

In the present systematic review, we report the existing literature regarding ASP implementation for antibiotic and antifungal management in pediatric oncology.

In the qualitative synthesis, we observed how ASPs could play a critical role in the management of infections in an oncology–hematology setting, with the main goal of optimizing antimicrobial administration. In particular, a reduction in the prescription of broad-spectrum antibiotics and an improved appropriateness were generally observed, with reduced antibiotic-related side effects and no difference in infection-related mortality.

Although stewardship strategies were different among the included studies, common types of interventions were found. Development or updating and dissemination of guidelines for the management of infections represents a key function of ASPs [14], and was included in most of papers. Education of physicians was also considered a main goal, pursued in different ways, ranging from periodic meetings to questionnaires distributed among pediatric oncologists. General objectives of ASPs include assessing prophylactic antimicrobial administration, reducing unnecessary antimicrobial initiation, and de-escalation of empirical therapy as soon as possible, including narrowing the spectrum, switching to oral therapy, and implementing discontinuation criteria. Implementation of diagnostic testing in order to better diagnose and monitor infections is considered another possible function of ASPs, in order to guide antimicrobial prescription.

An important aspect of ASPs consists of direct collaboration and discussion between the pediatric oncologists and the ASP team on antimicrobial therapy. The approach to this intervention should be modulated based on local policies [30]. Prior authorization of carbapenem prescription was implemented in one included study [23], and provides the benefit of directly reducing unnecessary antibiotic initiation, optimizing the selection of empirical antibiotics and encouraging infectious disease consultations. However, it could potentially delay antibiotic administration in critically ill patients, and could not address the subsequent antimicrobial management, such as intravenous-to-oral conversion or duration of therapy [14].

Auditing with feedback after antimicrobial prescription is a different strategy to apply antimicrobial stewardship, and was implemented clearly in three studies [22,23,24], while in one study a less clear post-prescription control was applied [29].

This approach has the advantage of greater flexibility in the timing of interventions, but does not affect initial prescription, and ASPs usually cannot enforce mandatory drug discontinuation [14]. In one study, both prior approval and post-prescription review were implemented [23], and we believe that this hybrid approach could represent the most effective way to implement ASPs [31].

In our qualitative synthesis, the measured outcomes in the different works were heterogeneous. Two studies reported a decrease in antibiotic use expressed as DOT per 1000 patient-days [23,25]. This metric is not affected by variations in dosing, accounts for the number of different antimicrobials administered [32], and could represent a useful parameter to better compare ASPs in future studies. To describe the effect of antimicrobial stewardship, and to enforce the implementation of ASPs among pediatric oncologists, safety analysis should be performed in order to assess the effects of antimicrobial prescription on clinical outcomes. It should be noted that none of the included studies reported differences in mortality or infection-related mortality after the implementation of ASPs, but further studies are needed to corroborate this finding. Among other clinical outcomes, adverse effects of antimicrobials and the incidence of multidrug-resistant microorganisms should be considered as important endpoints of ASPs. One paper found decreased *Clostridioides difficile* infections after ASP implementation [26], while two other studies reported reduced incidence of resistant strains [25,29]. Analysis of cost is essential for healthcare administrators, and ASP implementation has been associated with reduced antimicrobial-related expenditure in two settings [23,29]. This finding could help persuade administrators to allocate resources to ASPs.

Interestingly, the study by Dhanya et al. questioned the clinical relevance of colonization surveillance cultures to guide empirical antibiotic therapy in allo-HSCT [21]. This cohort of patients mostly included recipients of HSCT for hemoglobinopathies, in whom prior exposure to chemotherapy and intravenous (IV) antibiotics was not common. Thus, these findings might not be generalizable to patients undergoing transplantation for hematological malignancies. Monitoring of colonization by resistant strains represents a potentially useful strategy, as part of structured stewardship programs, and this is supported by several findings about the impact of MDR bacterial colonization on transplantation outcomes [33,34,35]. These partially conflicting results highlight the need for further studies in order to better understand the clinical relevance of colonization screening.

Of the nine included studies, four reported antifungal stewardship interventions. Several limitations could hinder the implementation of ASPs regarding antifungal prescription in pediatric oncology. There is a lack of clinical trials in children regarding diagnosis, prophylaxis, and treatment of fungal infections during anticancer therapies [27,36]. Difficulties in establishing a proven diagnosis and lower drug-related adverse effects in children also make the measurement of outcomes more difficult [28]. Furthermore, the epidemiology of *Candida* infections in children with cancers is changing, with an increase in *Candida* non-*albicans* [37]. Mortality associated with non-*albicans* species is higher; therefore, this trend represents a serious problem for the management of fungal infections [38,39]. Very interestingly, after the implementation of an ASP in the study of Amanati et al., non-*albicans* colonization was significantly reduced, with a significant decrease in C. *glabrata* colonization, as opposed to the trend observed in other institutions [37].

The main limitations of the qualitative synthesis provided in this study regard the paucity and heterogeneity of studies addressing this topic, in terms of both ASP strategies and measured outcomes (e.g., antibiotic use, clinical benefits, microbiological resistance, and costs). In particular, we found nine papers that matched our inclusion criteria. Two papers analyzed the implementation of ASPs, but lacked a pre-intervention phase comparison, leaving seven studies addressing the clinical and microbiological outcomes of antimicrobial stewardship interventions compared with a pre-intervention phase. We opted to extensively report only these studies in the tables. Moreover, these seven works report the results of single-center experiences.

## 5. Conclusions

In conclusion, the present systematic review shows how active antimicrobial stewardship interventions in pediatric oncology can reduce broad-spectrum antibiotic prescription and improve treatment appropriateness, with reduced antibiotic-related side effects and no difference in infection-related mortality. We observed that ASPs in pediatric oncology are implemented in heterogeneous ways between different institutions. Measurement of outcomes associated with antimicrobial stewardship interventions also differed in the included studies. We encourage future studies to implement DOT per 1000 patient-days as a method of monitoring antimicrobial use. Moreover, safety and cost analysis should be considered as two different but pivotal approaches in the study of ASP endpoints. In particular, we believe that monitoring antimicrobial ecology, with a particular focus on multidrug-resistant bacteria and *Candida* non-*albicans*, is a key component of ASPs.

Future approaches to be evaluated in antimicrobial stewardship include therapeutic drug monitoring (TDM) as a potential way to optimize antimicrobial efficacy while reducing toxicity [40], and the study of the impact of antibiotic selection on the gut microbiome, which has been shown to potentially modulate anticancer therapy’s adverse effects and outcomes [9,41,42,43].

## Figures and Tables

**Table 1:** Summary of the included studies regarding antibacterial stewardship reporting the comparison of clinical outcomes before and after the intervention.

First Author	Year	Type of Study	Antimicrobial Stewardship Interventions Applied	Results	Quality Assessment
Wattier [22]	2017	Observational study before and after the introduction of febrile neutropenia guidelines and antibacterial stewardship program; single center	Update of institutional guidelines on febrile neutropenia, particularly regarding the use of second-line Gram-negative agents (tobramycin and ciprofloxacin) Pediatric ASP conducted by an infectious disease doctor and pharmacist Prospective audit and feedback on inpatient antibiotics ASP teaching conference for oncology fellows	Tobramycin and ciprofloxacin use decreased significantly No change in anti-pseudomonal beta-lactam use, clinical outcomes (length of hospital stay, ICU admissions, in-hospital mortality), or Gram-negative isolates resistant to ciprofloxacin	Good
Horikoshi [23]	2018	Observational study before and after the introduction of antibacterial stewardship program; single center	Post-prescriptive review of carbapenem use Preauthorization of carbapenem Prospective audit with feedback Weekly meetings Consensus of FN management	No differences in appropriateness of bacteremia treatment (susceptibility of microorganisms isolated at blood culture to the empirical agent) Decreased use of antibiotics in DOT/1000 patient-days (cefepime, piperacillin/tazobactam, meropenem, vancomycin) Reduction in costs No differences in average hospital stay, all-cause mortality, or infection-related mortality	Poor
Hennig [24]	2017	Observational study before and after the introduction of febrile neutropenia guidelines and antibacterial stewardship program; single center	Updating of febrile neutropenia guidelines Education sessions Dissemination of written and verbal guidelines to medical staff Point-of-care antibiotic use feedback at weekly antimicrobial stewardship rounds and oncology rounds	Reduction in gentamicin use from 79% to 20% Increase in % of Gram-negative infections treated with gentamicin Decrease in % of infections without any growth in blood cultures treated with gentamicin Decrease in gentamicin administration > 48 h without TDM, and in gentamicin use without a Gram-negative isolate in blood culture > 48 h	Intermediate
Karandikar [25]	2019	Observational study before and after the introduction of febrile neutropenia guidelines; single center	Febrile neutropenia guidelines recommending intravenous vancomycin in all high-risk patients for 48 h at the beginning of the episode, with rapid discontinuation in the absence of positive blood culture or compatible clinical symptoms Updated antibiotic discontinuation criteria (appearing well, afebrile for >24 h, ANC > 200)	Overall and empirical antimicrobial DOT/1000 FN days decreased significantly for high-risk patients, from 2297 to 1758 and from 1388 to 973, respectively (*p* < 0.01) Overall and empirical vancomycin DOT/1000 FN days decreased from 311 to 166 and from 217 to 101, respectively (*p* < 0.01) Median empirical intravenous vancomycin days within an FN episode decreased significantly Incidence of VRE/1000 patient-days decreased from 2.53 to 0.90 (*p* = 0.002) No difference in 30-day all-cause mortality Increasing adherence to the protocol (for both standard and high-risk patients).	Good
Olson [26]	2020	Observational study before and after the implementation of guidelines concerning about home antibiotic use; single center	Guideline implementation recommending oral levofloxacin at discharge after febrile neutropenia in selected patients only	Decrease in home intravenous antibiotic use from 75% to 5% (*p* < 0.01) Increase in home oral levofloxacin use Decreased % of patients with intravenous antibiotic initiations within 24 h of a new healthcare encounter up to 7 days after discharge (from 12% to 4%) Decreased % of patients with blood culture collected within 7 days (from 9% to 4%) Longer length of hospital stay Higher median absolute neutrophil count at discharge Decrease in *C. difficile* testing and *C. difficile*-positive tests No differences in 30-day all-cause mortality	Intermediate

**Table 2:** Summary of included studies regarding antifungal stewardship reporting the comparison of clinical outcomes before and after the intervention.

First Author	Year	Type of Study	Antimicrobial Stewardship Interventions Applied	Results	Quality Assessment
Horikoshi [23]	2018	Observational study before and after the introduction of single-center antifungal stewardship program	Preauthorization of drug prescription Prospective audit with feedback by pharmacists and infectious disease specialists Ordering assistance for prescribing physicians Therapeutic drug monitoring by pharmacists Weekly clinical luncheon meeting of hematology–oncology and infectious disease physicians	Amphotericin B and fluconazole decreased significantly, by 75% and 41%, respectively Cost of antifungal agents decreased by 20%	Poor
Santiago-García [28]	2019	Observational study before and after the introduction of antifungal stewardship program; single center	Definition of a consensus protocol Training program held by an expert team of pediatric hematology–oncology and infectious disease specialists Self-assessment questionnaire distributed among physicians to assess their knowledge	Percentage of inadequate prescriptions decreased by 9.9% Acquired knowledge remained stable after 12 months in physicians	Intermediate
Amanati [29]	2021	Observational study before and after the introduction of antifungal stewardship program; single center	Actions aiming at learning, training, and continuous practice to improve the following fields: Appropriate treatment of the suspected IFDs Appropriate antifungal prescription Non-medical approach to prevent fungal infections	Reduction in *C. albicans* strains resistant to azoles, fluconazole, and caspofungin Reduction in fluconazole use Reduction in antifungal-related costs	Intermediate

## Data Availability

Not applicable.

## Supplementary Materials

The following supporting information can be downloaded at: https://www.mdpi.com/article/10.3390/jcm11154545/s1.
